# Invasive Ductal Carcinoma of the Breast Presenting With Contralateral Axillary Lymph Node Metastasis: A Case Report

**DOI:** 10.7759/cureus.69359

**Published:** 2024-09-13

**Authors:** Justin M Troelstra, Edward Murphy, Eleni Tousimis

**Affiliations:** 1 General Surgery, Cleveland Clinic Indian River Hospital, Vero Beach, USA; 2 Surgical Oncology, Cleveland Clinic Indian River Hospital, Vero Beach, USA

**Keywords:** breast cancer, breast oncology, contralateral axillary lymph node metastasis, general surgery and breast cancer, tumor staging

## Abstract

This is a case of a 71-year-old Caucasian female presenting with invasive carcinoma of the breast with contralateral axillary lymph node metastasis. This unique presentation presents clinicians with difficulty staging and, therefore, predicting patient prognosis. This patient had a history of right ER/PR positive HER2 negative stage 1 breast cancer s/p lumpectomy with sentinel lymph node biopsy and radiation plus tamoxifen x6 months in 2017. She is now presenting with a recurrence of right breast cancer along with additional metastatic disease to the contralateral (left) axilla. She was treated with a skin-sparing mastectomy along with a contralateral lymph node dissection. A negative sentinel lymph node was seen, representing the dilemma of non-contiguous metastatic spread. Histological pathology of the right breast masses revealed multifocal invasive carcinoma of no special type (ductal), along with two of five left axillary lymph nodes positive for metastatic mammary carcinoma. No right-sided sentinel node was identified. The right breast lesions and the left axillary lymph node metastases are all morphologically similar and showed strong ER expression, the results of which are compatible with spreading to the contralateral axilla.

## Introduction

Breast cancer is the leading malignancy diagnosed worldwide and is the leading cause of cancer death in women globally [1]. With the emergence of improved breast cancer screening techniques, the mortality rate for breast cancers has been declining steadily since the 1970s. This phenomenon was represented in a landmark article revealing that women who participated in organized mammography screening had a 60% lower risk of dying of breast cancer within 10 years of diagnosis [2]. The mortality rate decline can also be attributed to more specific and targeted therapies, decreasing the burden of systemic toxicities. After the diagnosis of breast cancer is confirmed via biopsy, treatment recommendations depend on the extent of the disease, including but not limited to histopathology of the tumor, receptor expression, extent of lymph node involvement, as well as the presence of metastasis.

The tumor, node, metastasis (TNM) staging system of breast cancer is an internationally accepted system used to determine the severity of disease along with guiding prognosis and treatment. Recent updates to the TNM staging system include biological markers, which improve discrimination of prognosis when compared to anatomic staging alone. Important to consider after the diagnosis of breast cancer is the preoperative examination of ipsilateral lymph nodes, as it guides perioperative treatment. Patients with clinically positive nodes typically undergo axillary lymph node dissection, while patients with clinically negative disease often undergo sentinel lymph node biopsy perioperatively [3]. In the case of contralateral lymph node-positive disease, as in this case, the TNM classification is not a clear picture, thus making treatment and prognosis clinically difficult. This presentation is infrequent and presents a dilemma for clinicians [4].

## Case presentation

This patient is a 71-year-old Caucasian female who presented after noticing a right breast mass over the preceding month. The patient had a history of right-stage I breast cancer status post lumpectomy with sentinel lymph node biopsy and radiation therapy in 2017. She reportedly lumped her baseline secondary to scar tissue from her previous lumpectomy. She had a hematoma drained after her previous lumpectomy, most likely leading to a delay in seeking medical care. Palpation of the right axilla was negative for any masses or lumps. A diagnostic mammogram was performed that revealed a 15 x 9.35 mm irregularly shaped, micro-lobulated, hypoechoic mass with associated calcification. In the left axilla, a 15 x 11 x 19 mm lymph node with cortical thickening was also visualized. These radiographic findings coincided with a BIRAD classification of 5, which is highly suggestive of malignancy (95% or greater), as represented in Table 1. The BIRADs classification system standardizes the reporting of mammographic findings and their coinciding recommendations for further management. An important note to consider with regard to this type of classification is that it does not consider the clinical picture; therefore, a clinically specific lump without an alarming finding on imaging may still warrant further examination with a biopsy [5].

**Table 1:** BIRADs classification system is used to stratify patients on likelihood of malignancy depending upon imaging characteristics.

Assessment	Management	Likelihood of Cancer
Category 0: Incomplete. Need additional imaging evaluation and/or prior mammograms	Recall for additional imaging and/or comparison with prior examination(s)	N/a
Category 1: Negative	Routine mammography screening	Essentially 0% likelihood of malignancy
Category 2: Benign	Routine mammography screening	Essentially 0% likelihood of malignancy
Category 3: Probably benign	Short-interval (six-month) follow-up or continued surveillance mammography	>0% but <2% likelihood of malignancy
Category 4: Suspicious	Tissue diagnosis	>0% but <2% likelihood of malignancy
Category 4A: Low suspicion for malignancy	Tissue diagnosis	>2 but <95% likelihood of malignancy
Category 4B: Moderate suspicious for malignancy	Tissue diagnosis	>2 to <10% likelihood of malignancy
Category 4C: High suspicion for malignancy	Tissue diagnosis	>10 to <50% likelihood of malignancy
Category 5: Highly suggestive of malignancy	Tissue diagnosis	>50 to <95% likelihood of malignancy
Category 6: Known biopsy-proven malignancy	Surgical Excision when clinically proven	>95% likelihood of malignancy

**Figure 1:**
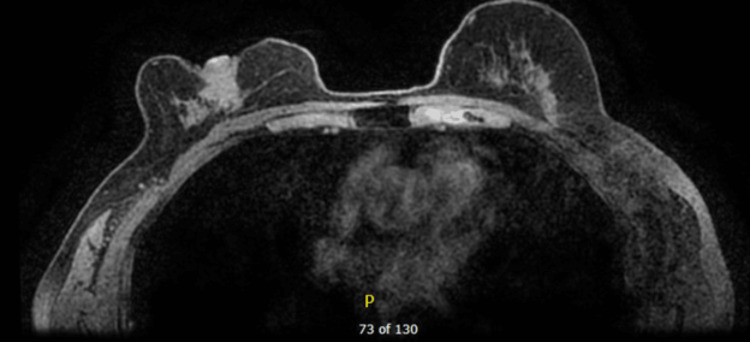
MRI of the right breast mass for further evaluation in order to guide further management.

**Figure 2:**
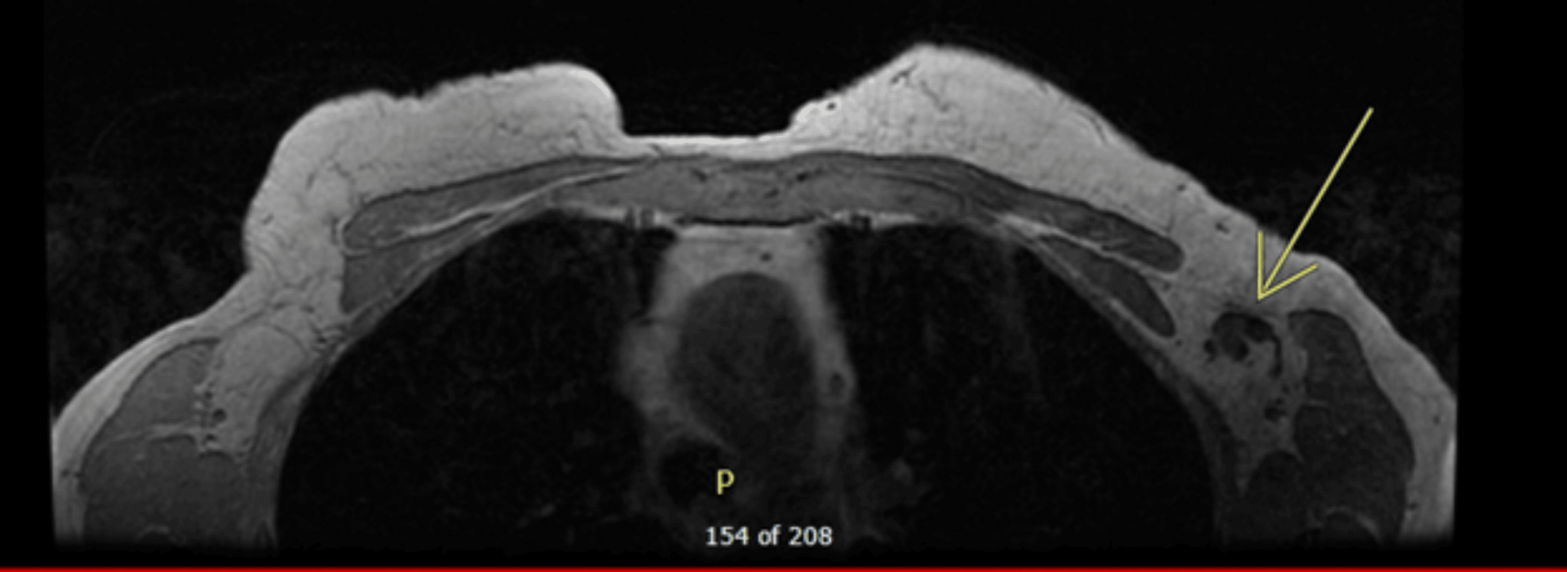
MRI of the left axillary lymph node for further evaluation in order to guide further management.

Following the diagnostic mammogram, a bilateral MRI (magnetic resonance imaging) was obtained due to multiple diagnoses. On MRI, an additional 2.5 x 1.3 x 1.2 cm bilobed-shaped lobulated mass was also seen immediately inferior to the above-described mass. No abnormalities were seen in the right axillary and internal mammary lymph nodes. The left breast did not reveal any suspicious mass or enhancement; however, a 1.5 cm left axillary lymph node with a thickened cortex was visualized. These MRI findings can be seen below in Figures 1, 2.

A needle core biopsy was performed of the right breast masses that revealed malignant invasive ductal carcinoma and rare ductal carcinoma, respectively. A needle core biopsy of the left axillary lymph node was also performed, revealing 1 lymph node with metastatic, estrogen receptor-positive carcinoma consistent with metastatic breast carcinoma. Magseed (Endomagnetics, Ltd.) clips were then placed in the left axillary lymph node mass via ultrasound (US) guidance. The patient then proceeded to undergo a right skin-sparing mastectomy along with a left axillary lymph node dissection after Magseed localization. The sentinel lymph node probe was used to find the right sentinel lymph node, and there was no focal signal in the patient’s right axilla. The right axilla was also palpated, which did not reveal any evidence of disease. The left axillary lymph node was identified with the Magseed probe, and the nodes were dissected free. The specimens were sent to pathology for a permanent section. Histological pathology of the right breast masses revealed multifocal invasive carcinoma of no special type (ductal) along with two of five left axillary lymph nodes positive for metastatic mammary carcinoma. Masses were ER/PR positive and HER2 FISH negative, oncotypes 19 and 14. No right-sided sentinel node was identified. The right breast lesions and the left axillary lymph node metastases were all morphologically similar and showed strong ER expression, results that are compatible with spreading to the contralateral axilla.

Post-operatively, the patient recovered well without complications. A PET scan at the time of diagnosis did not reveal any evidence of distant metastasis. Her case was discussed at the breast cancer tumor board, where it was determined that she would benefit from post-operative radiation therapy, which she has now completed. The patient is currently taking Anastrozole 1 mg without side effects. It was also discussed with the patient that she is to begin Abemaciclib 100 mg twice daily at the end of radiation. Her radiation included 4256 centigray (cGy)/16 fractions. She was instructed of the expected adverse effects, and her complete blood count is to be monitored initially every 2 weeks and then once a month. The patient is in agreement and amenable to the stated plan. Of note, the patient is from the Midwest and plans to return there for the summer months and therefore will resume medical oncologic follow-up there. She plans on returning to Florida in the fall, at which restaging scans will be repeated and subsequently discussed with her at a follow-up appointment.

## Discussion

Given the prevalence of breast cancer both worldwide and in the United States, optimizing screening, diagnosis, and treatment is important for adequate patient care. This case represents a difficult clinical dilemma as no (left) ipsilateral primary breast mass was found, thus making stage IV metastatic breast cancer the most likely diagnosis. The large differences in clinical management of breast cancer derive from the stratification of patients into “early-stage breast cancer” versus “locally advanced” versus metastatic breast cancer.

Early-stage breast cancer is most often treated with either a lumpectomy or mastectomy with or without radiation therapy. The decision to undergo breast-conserving therapy versus complete mastectomy has been shown to have equivalent disease-free and overall survival [6-9]. In locally advanced breast cancer, patients most often undergo neoadjuvant therapy to induce a tumor response before surgery to hopefully be able to allow for breast conservation. Lastly, in metastatic breast cancer, treatment is least likely to be curative; however, advancements in treatment have prolonged survival.

The role of a sentinel lymph node biopsy (SLND) in clinically negative lymph nodes has favorable morbidity benefits for patients. First off, it allows for the early staging of the malignancy. It also is less invasive and does not require a full axillary lymph node dissection, which comes with an increased risk of lymphedema. Additionally, if a SLNB is negative, it negates the need for additional surgery to remove more lymph nodes. 

Upon diagnosis of metastatic breast cancer, hormone receptor and human epidermal growth factor receptor 2 (HER2) status should be repeated. Germline breast cancer susceptibility genes should also be assessed, as these factors guide treatment regimens. In patients with triple-negative disease (estrogen receptor (ER) negative, progesterone receptor (PR) negative, and HER2 negative), the cancer is presumably an aggressive subtype, and thus first-line chemotherapy is recommended. Receptor-positive diseases, however, represent less aggressive tumors, and therefore more specific targeted therapies are available [10].

## Conclusions

As previously stated, this patient’s presentation represents a clinically difficult diagnosis, particularly in the staging of breast cancer, and thus prognosis and treatment. Given that the prevalence of contralateral axillary lymph node metastasis is a rare occurrence, the steps to approach this diagnosis are not well defined. Taking into account the clinical scenario, as well as imaging findings, allows for risk stratification to provide the most efficacious treatment strategies to minimize morbidity and maximize the mortality benefit.
